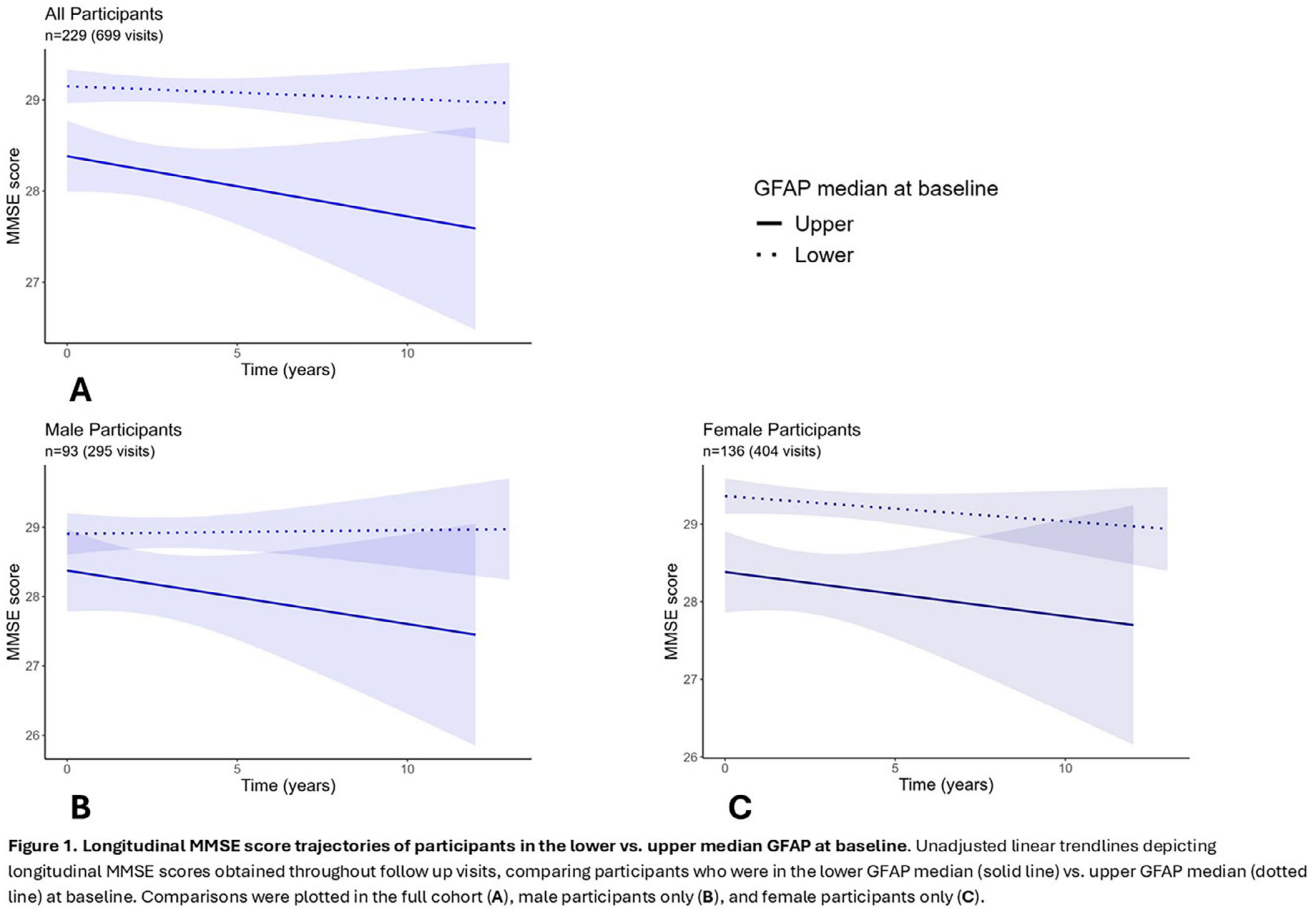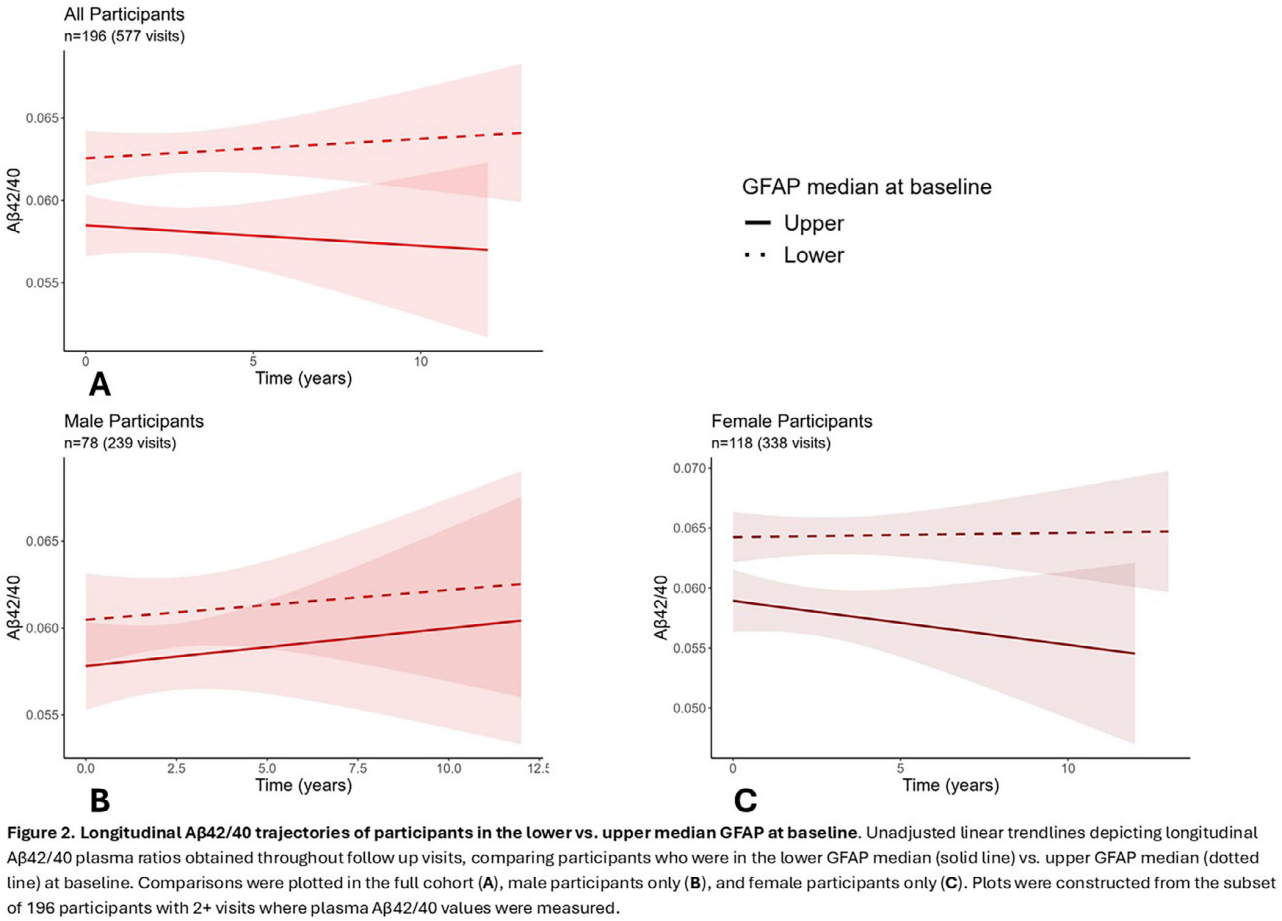# Sex‐Differences in Association between Plasma GFAP and Cognition in Cognitively Unimpaired Community Dwelling Older Adults

**DOI:** 10.1002/alz70856_107006

**Published:** 2026-01-07

**Authors:** Chelsea Reichert Plaska, Tovia Jacobs, Davide Bruno, Katherine Brundage, Sang Han Lee, Jaime Ramos Cejudo, Ricardo S. Osorio, Bruno P Imbimbo, Nicholas J. Ashton, Henrik Zetterberg, Kaj Blennow

**Affiliations:** ^1^ NYU Grossman School of Medicine, New York, NY, USA; ^2^ Nathan S. Kline Institute, Orangeburg, NY, USA; ^3^ Liverpool John Moores University, Liverpool, United Kingdom; ^4^ Rush University Medical Center, Chicago, IL, USA; ^5^ Ferkauf School of Psychology, Yeshiva University, Bronx, NY, USA; ^6^ Chiesi Farmaceutici, Parma, Italy; ^7^ Department of Psychiatry and Neurochemistry, Institute of Neuroscience & Physiology, the Sahlgrenska Academy at the University of Gothenburg, Mölndal, Sweden; ^8^ Hong Kong Center for Neurodegenerative Diseases, Hong Kong, Science Park, China; ^9^ Clinical Neurochemistry Laboratory, Sahlgrenska University Hospital, Mölndal, Västra Götalands län, Sweden; ^10^ Department of Neurodegenerative Disease, UCL Institute of Neurology, London, United Kingdom; ^11^ Wisconsin Alzheimer's Disease Research Center, University of Wisconsin‐Madison, School of Medicine and Public Health, Madison, WI, USA; ^12^ Institute of Neuroscience and Physiology, Sahlgrenska Academy at the University of Gothenburg, Gothenburg, Sweden; ^13^ UK Dementia Research Institute at UCL, London, United Kingdom; ^14^ Department of Psychiatry and Neurochemistry, Institute of Neuroscience and Physiology, The Sahlgrenska Academy, University of Gothenburg, Mölndal, Sweden; ^15^ Clinical Neurochemistry Laboratory, Sahlgrenska University Hospital, Mölndal, Sweden

## Abstract

**Background:**

Women are at greater risk of developing sporadic Alzheimer's disease (AD). While their longer lifespans contribute to AD‐risk, this factor does not fully explain the observed sex differences. Increased glial activation and neuroinflammation associated with female‐sex may play a role. Glial fibrillary acidic protein (GFAP), a marker of astrocyte activation and neuroinflammation, has been linked to AD‐ risk. In preclinical and prodromal AD, higher brain GFAP levels are associated with cognitive decline, Aβ deposition, tau spread, and neurodegeneration associated with AD. Studies have reported that plasma GFAP levels are higher in women compared to men, but few have thoroughly examined this influence. This prompted us to examine sex‐differences in plasma GFAP in cognitively unimpaired older adults and its relationship to cognitive performance and other plasma AD biomarkers, both at baseline and longitudinally.

**Method:**

Participants were enrolled in the Memory Education and Research Initiative (MERI) program and completed a neuropsychological battery, clinical and psychiatric evaluation, and blood draw. Plasma GFAP, Aβ42, Aβ40 concentrations were measured using single‐molecule array (SIMOA) platform. Participants were included if they gave blood (2+ timepoints), MMSE>23 at Baseline, and age 50‐85. Analysis of covariance (ANCOVA) was conducted to assess for sex differences, and longitudinal analyses using linear mixed models were conducted to examine associations with cognition.

**Result:**

A total 325 MERI (mean age=72 years; 59% females) were included in the analysis. ANCOVA, controlling for age, showed significantly higher plasma GFAP levels in females compared to males (*p* = 0.001). Longitudinal analysis of 229 MERI revealed that higher baseline GFAP is associated with lower MMSE scores over time (Figure 1, *p* = 0.002). When stratified by sex, these associations remained significant in females but not males (*p* = 0.002). GFAP was also inversely associated with Aβ42/40 over time (Figure 2, *p* <0.001) in females.

**Conclusion:**

Cognitively unimpaired females had higher levels of plasma GFAP compared to males. Moreover, elevated GFAP levels associated with greater decline in global cognition and lower Aβ42/40 in females but not males. These findings suggest increased astrocytic activation that may lead to cognitive decline in females. More studies are needed to understand how sex‐differences in neuroinflammation may impact future AD‐risk. Potential mechanisms will be discussed.